# Constraints on tensor and scalar couplings from $B\rightarrow K\bar{\mu }\mu$ and $B_s\rightarrow \bar{\mu }\mu$

**DOI:** 10.1140/epjc/s10052-015-3676-2

**Published:** 2015-09-28

**Authors:** Frederik Beaujean, Christoph Bobeth, Stephan Jahn

**Affiliations:** C2PAP, Excellence Cluster Universe, Ludwig-Maximilians-Universität München, Garching, Germany; Institute for Advanced Study, Technische Universität München, Garching, Germany; Excellence Cluster Universe, Technische Universität München, Garching, Germany; Max Planck Institute for Physics, Munich, Germany

## Abstract

The angular distribution of  ($\ell = e,\,\mu ,\,\tau$) depends on two parameters, the lepton forward–backward asymmetry, $A_\mathrm{FB}^\ell$, and the flat term, $F_H^\ell$. Both are strongly suppressed in the standard model and constitute sensitive probes of tensor and scalar contributions. We use the latest experimental results for $\ell = \mu$ in combination with the branching ratio of $B_s\rightarrow \bar{\mu }\mu$ to derive the strongest model-independent bounds on tensor and scalar effective couplings to date. The measurement of $F_H^\mu$ provides a complementary constraint to that of the branching ratio of $B_s\rightarrow \bar{\mu }\mu$ and allows us – for the first time – to constrain all complex-valued (pseudo-)scalar couplings and their chirality-flipped counterparts in one fit. Based on Bayesian fits of various scenarios, we find that our bounds even become tighter when vector couplings are allowed to deviate from the standard model and that specific combinations of angular observables in $B \rightarrow K^*\bar{\ell }\ell$ are still allowed to be up to two orders of magnitude larger than in the standard model, which would place them in the region of LHCb’s sensitivity.

## Introduction

With the analysis of the data collected by the LHCb Collaboration during run I at the Large Hadron Collider (LHC), we now have access to rather large samples of rare *B*-meson decays with branching ratios below $10^{-5}$. As a consequence, angular analyses of three- and four-body final states can be used to measure a larger number of observables than previously possible at the B factories BaBar and Belle. In this work we focus on rare *B* decays driven at the parton level by the flavor-changing neutral-current (FCNC) transition $b\rightarrow s \bar{\ell }\ell$ that constitutes a valuable probe of the standard model (SM) and provides constraints on its extensions.

The angular distribution of $B\rightarrow K \bar{\ell }\ell$ – normalized to the width $\Gamma _\ell$ – in the angle $\theta _\ell$ between *B* and $\ell ^-$ as measured in the dilepton rest frame is1.1LHCb analyzed their full run 1 data set of 3 fb$^{-1}$ and measured the angular distribution of the mode $B^+\rightarrow K^+ \bar{\mu }\mu$, i.e. $\ell = \mu$ [1], with unprecedented precision. They provide the lepton forward–backward asymmetry $A_\mathrm{FB}^\mu$ and the flat term $F_H^\mu$ in CP-averaged form and integrated over several bins in the dilepton invariant mass $q^2$. Similarly, the CP-averaged branching ratios, ${\mathscr {B}}_\mu = \tau _{B} \Gamma _\mu$, [2] and the rate CP asymmetry $A_\mathrm{CP}^\mu$ [3] are also available from 3 fb$^{-1}$.

Both angular observables, $F_H^\ell$ and $A_\mathrm{FB}^\ell$, exhibit strong suppression factors for vector and dipole couplings present in the SM, thereby enhancing their sensitivity to tensor and scalar couplings [4, 5]. A similar enhancement of scalar couplings compared to helicity-suppressed vector couplings of the SM is well known from $B_s\rightarrow \bar{\mu }\mu$. Unfortunately the limited data set of $B\rightarrow K^*(\rightarrow K\pi ) \bar{\ell }\ell$ from LHCb [6] did not yet allow one to perform a full angular analysis without the assumption of vanishing scalar and tensor couplings in this decay mode. In the future with more data or special-purpose analysis techniques like the method of moments [7], certain angular observables in $B\rightarrow K^* \bar{\ell }\ell$ will provide additional constraints on such couplings, as for example $J_{6c}$ [8] and the linear combinations $(J_{1s} - 3 J_{2s})$ and $(J_{1c} + J_{2c})$ [5, 9] as well as the experimental test of the relations $H_T^{(2)} = H_T^{(3)}$ and $J_7 = 0$ [5] at low hadronic recoil.

Here we exploit current data from $B^+\rightarrow K^+ \bar{\mu }\mu$ and $B_s\rightarrow \bar{\mu }\mu$ to derive stronger constraints than before on tensor and scalar couplings in various model-independent scenarios and study their impact on the not-yet-measured sensitive observables in $B\rightarrow K^* \bar{\ell }\ell$. In Sect. 2, we specify the effective theory of $|\Delta B| = 1$ decays on which our model-independent fits are based. Within this theory, we discuss the dependence of observables in $B\rightarrow K \bar{\ell }\ell$ and $B\rightarrow K^* \bar{\ell }\ell$ on the tensor and scalar couplings in Sect. 3 and specify also the experimental input used in the fits. The constraints on tensor and scalar couplings from the data are presented for several model-independent scenarios in Sect. 4. Technical details of the angular observables in $B\rightarrow K^* \bar{\ell }\ell$, the branching fraction of $B_s\rightarrow \bar{\mu }\mu$, the treatment of theory uncertainties, and the Monte Carlo methods used are relegated to appendices.

## Effective theory

In the framework of the $|\Delta B| = |\Delta S| = 1$ effective theory

2.1 $$\begin{aligned} {\mathscr {L}}_\mathrm{eff}&= \frac{4 G_{F}}{\sqrt{2}} \,\frac{\alpha _e}{4 \pi } V_{tb}^{} V_{ts}^*\, \bigg [ {C_7(\mu _b) {\mathscr {O}}_7 + C_{7'}(\mu _b) {\mathscr {O}}_{7'}} \nonumber \\&\quad + \sum _{\ell = e,\, \mu ,\, \tau } \sum _i \mathscr {C}_{i}^{\mathrm {\ell }}(\mu _b) {{\mathscr {O}}_i^\ell }\bigg ] + \text {h.c.}, \end{aligned}$$

the most general dimension-six flavor-changing operators ${\mathscr {O}}_i^{(\ell )}$ mediating $b\rightarrow s \gamma$ and $b\rightarrow s \bar{\ell }\ell$ are classified according to their chiral structure. There are dipole ($i = 7,7'$) and vector ($i = 9,9',10,10'$) operators,

2.2 $$\begin{aligned}&{\mathscr {O}}_{7(7')} = \frac{m_b}{e} \left[ \bar{s} \sigma ^{\mu \nu } P_{R(L)} b \right] F_{\mu \nu },\nonumber \\&{{\mathscr {O}}_{9(9')}^\ell } = \left[ \bar{s} \gamma _\mu P_{L(R)} b \right] \left[ \bar{\ell } \gamma ^\mu \ell \right] , \nonumber \\&{{\mathscr {O}}_{10(10')}^\ell } = \left[ \bar{s} \gamma _\mu P_{L(R)} b\right] \left[ \bar{\ell } \gamma ^\mu \gamma _5 \ell \right] , \end{aligned}$$

further scalar ($i = S,S',P,P'$) operators,

2.3 $$\begin{aligned}&{{\mathscr {O}}_{S(S')}^\ell } = \left[ \bar{s} P_{R(L)} b \right] \left[ \bar{\ell } \ell \right] ,\nonumber \\&{{\mathscr {O}}_{P(P')}^\ell } = \left[ \bar{s} P_{R(L)} b\right] \left[ \bar{\ell } \gamma _5 \ell \right] , \end{aligned}$$

and tensor ($i = T,T5$) operators,

2.4 $$\begin{aligned}&{{\mathscr {O}}_{T}^\ell } = \left[ \bar{s} \sigma _{\mu \nu } b\right] \left[ \bar{\ell } \sigma ^{\mu \nu } \ell \right] ,\nonumber \\&{{\mathscr {O}}_{T5}^\ell } = \left[ \bar{s} \sigma _{\mu \nu } b\right] \left[ \bar{\ell } \sigma ^{\mu \nu } \gamma _5 \ell \right] , \end{aligned}$$

where the notation ${{\mathscr {O}}_{T5}^\ell } = i/2\, \varepsilon ^{\mu \nu \alpha \beta } [\bar{s} \sigma _{\mu \nu } b] [\bar{\ell } \sigma _{\alpha \beta } \ell ]$ is also used frequently in the literature. The respective short-distance couplings, the Wilson coefficients $\mathscr {C}_{i}^{\mathrm {(\ell )}}(\mu _b)$, are evaluated at a scale of the order of the *b*-quark mass $\mu _b \sim m_b$ and can be modified from SM predictions in the presence of new physics.

The SM values $\mathscr {C}_{7,9,10}^{\mathrm {(\ell )}}$ are obtained at next-to-next-to leading order (NNLO) [10, 11] and depend on the fundamental parameters of the top-quark and *W*-boson masses, as well as on the sine of the weak mixing angle. Moreover, they are universal for the three lepton flavors $\ell = e,\, \mu ,\, \tau$. All other Wilson coefficients are numerically suppressed or zero: $\mathscr {C}_{7'}^{\mathrm {SM}} = m_s/m_b\, \mathscr {C}_{7}^{\mathrm {SM}}$, $\mathscr {C}_{S,S',P,P'}^{\mathrm {\ell , SM}} \sim {m_b m_\ell /m_W^2}$, and $\mathscr {C}_{9',10',T,T5}^{\mathrm {\ell , SM}} = 0$. The Wilson coefficients of the four-quark current–current and QCD-penguin operators as well as of the chromomagnetic dipole operators are set to their NNLO SM values at $\mu _b = 4.2 \, \text{ GeV }$ [10, 11].

For the rest of this article, we will suppress the lepton-flavor index on the Wilson coefficients $\mathscr {C}_{i}^{\mathrm {\ell }} \rightarrow \mathscr {C}_{i}^{\mathrm {}}$ and operators ${\mathscr {O}}_i^\ell \rightarrow {\mathscr {O}}_i$. In Sect. 4 we exploit data with $\ell =\mu$ only, hence all derived constraints apply in principle only to the muonic case but can be carried over to the other lepton flavors $\ell = e,\, \tau$ for NP models that do not violate lepton flavor. In general, the Wilson coefficients are decomposed into SM and NP contributions $\mathscr {C}_{i}^{\mathrm {}} = \mathscr {C}_{i}^{\mathrm {SM}} + \mathscr {C}_{i}^{\mathrm {NP}}$ but often we will use $\mathscr {C}_{i}^{\mathrm {}}$ for Wilson coefficients with zero (or suppressed) SM contributions synonymously with $\mathscr {C}_{i}^{\mathrm {NP}}$.

## Observables and experimental input

The full dependence of $F_H^\ell$ and $A_\mathrm{FB}^\ell$ on tensor and scalar couplings has been presented in [4, 5], adopting the effective theory (2.1), i.e. neglecting higher-dimensional operators with $\text{ dim } \ge 8$. These results imply that for SM values of the effective couplings3.1Hence, for $\ell = e,\, \mu$ both observables are quasi-null tests. The flat term $F_H^\ell (q^2)|_\mathrm{SM}$ is strongly suppressed by small lepton masses for the considered kinematic region $1 \le q^2 \le 22$ GeV$^2$ [4, 12, 13]. Nonzero values of $A_\mathrm{FB}^\ell |_\mathrm{SM}$ can be induced by higher-order QED corrections, which will modify the simple $\cos \theta _\ell$ dependence of the angular distribution (1.1); however, currently there is no solid estimate available for this source of SM background.^1^ This picture does not change in the presence of new-physics contributions to vector and dipole operators $i = 7,7',9,9',10,10'$. On the other hand, nonvanishing tensor or scalar contributions are enhanced unless the dynamics of NP implies similar suppression factors, i.e., lepton–Yukawa couplings for $F_H^\ell$ or $\alpha _e$ in the case of $A_\mathrm{FB}^\ell$. In particular, $F_H^\ell$ is very sensitive to tensor couplings (see (3.3) below) and $A_\mathrm{FB}^\ell$ is sensitive to the interference of tensor and scalar couplings (see (3.6) below).

**Fig. 1:**
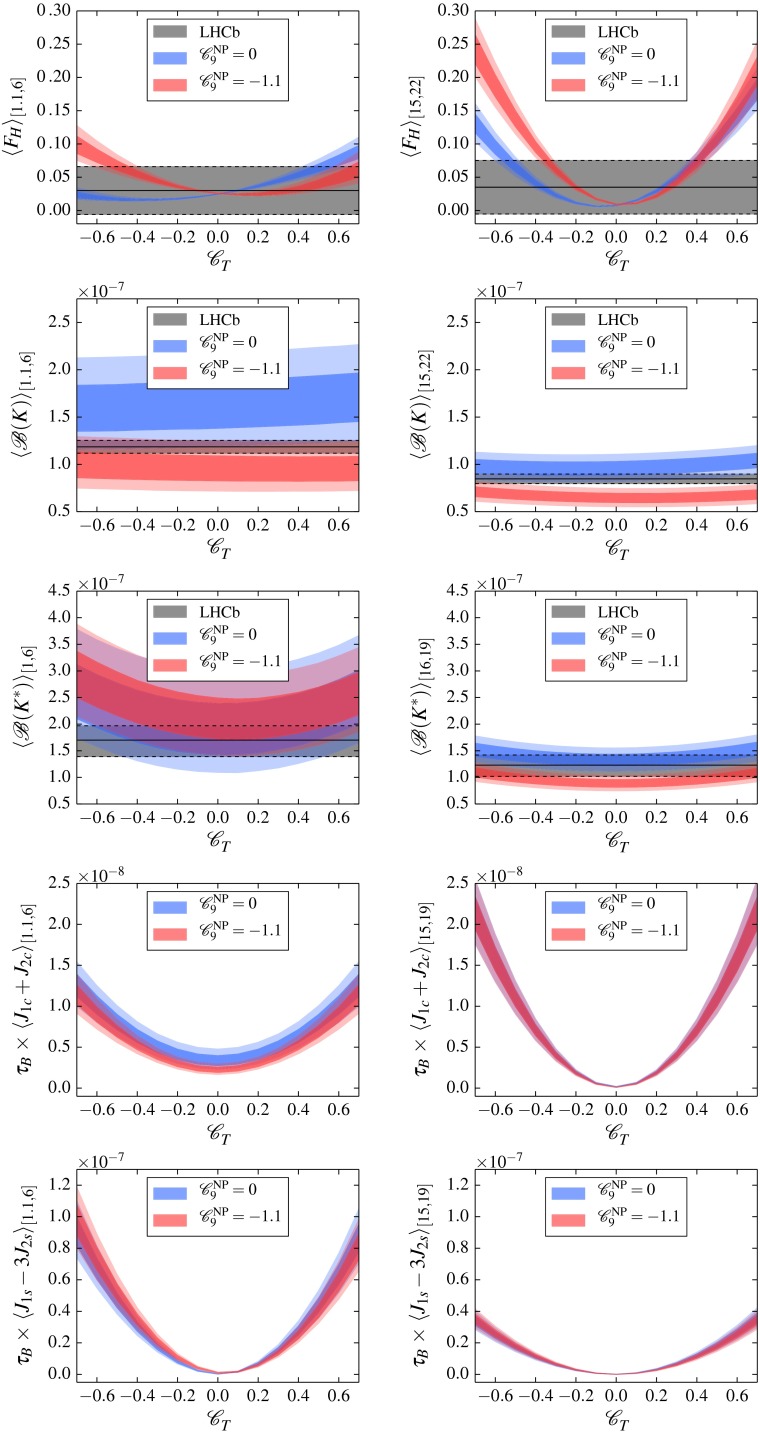
The sensitivity to the tensor coupling $\text{ Re }(\mathscr {C}_{T}^{\mathrm {}})$ of $F_H^\mu$ and ${\mathscr {B}}_\mu$ in $B^+ \rightarrow K^+ \bar{\mu }\mu$ as well as ${\mathscr {B}}_\mu$, $(J_{1c} + J_{2c})$, and $(J_{1s}-3 J_{2s})$ in $B^0 \rightarrow K^{*0} \bar{\mu }\mu$. Angular observables are rescaled by the lifetime of the *B* meson, $\tau _B$. The *bands* represent the theory uncertainties at 68 and 95 % probability of the prior predictive. Two sets of bands are shown for $\mathscr {C}_{9}^{\mathrm {NP}} = 0$ (*blue*) and $\mathscr {C}_{9}^{\mathrm {NP}} = -1.1$ (*red*). If available, the *gray band* indicates the latest 68 % confidence interval reported by LHCb. All observables are integrated over $q^2 \in [q_1^2,\,q_2^2]$ bins denoted as $\langle \dots \rangle _{[q^2_1,\, q^2_2]}$ to match LHCb

There are some angular observables $J_i$ in $B\rightarrow K^*(\rightarrow K\pi )\, \bar{\ell }\ell$ with the same properties; i.e. tensor and scalar contributions are kinematically enhanced by a factor $\sqrt{q^2}/m_\ell$ over vector ones present in the SM or their respective interference terms. These are $J_{6c}$ and the two linear combinations $(J_{1s} - 3 J_{2s})$ and $(J_{1c} + J_{2c})$ with explicit formulas given in Appendix A. In our fits and predictions we include all kinematically suppressed terms. But for the purpose of illustration, we now consider the analytical dependence for vanishing lepton mass. In this limit,

3.2 $$\begin{aligned} J_{6c}&\propto \text{ Re } \left[ (\mathscr {C}_{P}^{\mathrm {}} - \mathscr {C}_{P'}^{\mathrm {}})\, \mathscr {C}_{T}^{\mathrm {*}} - (\mathscr {C}_{S}^{\mathrm {}} - \mathscr {C}_{S'}^{\mathrm {}})\, \mathscr {C}_{T5}^{\mathrm {*}}\right] \end{aligned}$$

is sensitive to the interference of tensor and scalar operators, complementary to $A_\mathrm{FB}^\ell$ in $B\rightarrow K \bar{\ell }\ell$

3.3 $$\begin{aligned} A_\mathrm{FB}^\ell&\propto \text{ Re } \left[ (\mathscr {C}_{P}^{\mathrm {}} + \mathscr {C}_{P'}^{\mathrm {}})\, \mathscr {C}_{T5}^{\mathrm {*}} + (\mathscr {C}_{S}^{\mathrm {}} + \mathscr {C}_{S'}^{\mathrm {}})\, \mathscr {C}_{T}^{\mathrm {*}}\right] \Big / \Gamma _\ell , \end{aligned}$$

i.e., with an interchange of tensor contributions $T \leftrightarrow T5$. We note also that $J_{6c}$ contributes to the lepton forward–backward asymmetry of $B\rightarrow K^* \bar{\ell }\ell$ being $\propto (J_{6s} + J_{6c}/2)$. Since it has to compete with $J_{6s}$ in this observable, a separate measurement of $J_{6s}$ and $J_{6c}$ is necessary.

Only tensor contributions enter

3.4 $$\begin{aligned} (J_{1s} - 3\, J_{2s})&\propto \left( \ldots |\mathscr {C}_{T}^{\mathrm {}}|^2 + \cdots |\mathscr {C}_{T5}^{\mathrm {}}|^2 \right) , \end{aligned}$$

where the dots indicate different kinematic and form-factor dependencies. But tensor *and* scalar contributions enter

3.5 $$\begin{aligned} (J_{1c} + J_{2c})&\propto \cdots \left( |\mathscr {C}_{T}^{\mathrm {}}|^2 + |\mathscr {C}_{T5}^{\mathrm {}}|^2 \right) \nonumber \\&\quad + \cdots \left( |\mathscr {C}_{S}^{\mathrm {}} - \mathscr {C}_{S'}^{\mathrm {}}|^2 + |\mathscr {C}_{P}^{\mathrm {}} - \mathscr {C}_{P'}^{\mathrm {}}|^2 \right) , \end{aligned}$$

which is similar to the dependence of $F_H^\ell$ in $B\rightarrow K \bar{\ell }\ell$

3.6 $$\begin{aligned} F_H^\ell \propto&\left[ \cdots \left( |\mathscr {C}_{T}^{\mathrm {}}|^2 + |\mathscr {C}_{T5}^{\mathrm {}}|^2\right) \right. \nonumber \\&\quad \left. + \cdots \left( |\mathscr {C}_{S}^{\mathrm {}} + \mathscr {C}_{S'}^{\mathrm {}}|^2 + |\mathscr {C}_{P}^{\mathrm {}} + \mathscr {C}_{P'}^{\mathrm {}}|^2 \right) \right] \Big / \Gamma _\ell . \end{aligned}$$

Concerning $F_H^\ell$, the involved kinematic factors – see [4, 5] – are such that tensor and scalar couplings contribute only constructively/cumulatively, apart from cancellations among $\mathscr {C}_{S(P)}^{\mathrm {}}$ and $\mathscr {C}_{S'(P')}^{\mathrm {}}$. Interference terms in the numerator of $F_H^\ell$ of the form $(\mathscr {C}_{T}^{\mathrm {}} \times \mathscr {C}_{7,7',9,9'}^{\mathrm {}})$ and $(\mathscr {C}_{P,P'}^{\mathrm {}} \times \mathscr {C}_{10,10'}^{\mathrm {}})$ are suppressed by $m_\ell /\sqrt{q^2}$. They become numerically relevant in case $\mathscr {C}_{T}^{\mathrm {}} \ll \mathscr {C}_{7,7',9,9'}^{\mathrm {}}$ or $\mathscr {C}_{P,P'}^{\mathrm {}} \ll \mathscr {C}_{10,10'}^{\mathrm {}}$ where the smallness of $\mathscr {C}_{T,P,P'}^{\mathrm {}}$ is of the same level as the suppression factor $m_\ell /\sqrt{q^2}$ accompanying the large vectorial SM Wilson coefficients $\mathscr {C}_{9,10}^{\mathrm {SM}} \sim \pm 4$. This implies, however, no large enhancement of $F_H^\ell$ over the SM prediction.

On the one hand, the observables $F_H^\ell$ (3.6) and $A_\mathrm{FB}^\ell$ (3.3) are measured in the angular distribution (1.1) of $B\rightarrow K \bar{\ell }\ell$ normalized to the decay width $\Gamma _\ell$ such that uncertainties due to form factors can cancel in part [4, 5]. On the other hand, $J_{6c}$, $(J_{1s} - 3\, J_{2s})$, and $(J_{1c} + J_{2c})$ appear in the unnormalized angular distribution of $B\rightarrow K^* \bar{\ell }\ell$. “Optimized” versions $S_1$, $M_1$, and $M_2$ for the low-$q^2$ region for which form factors cancel in the limit of $m_b \rightarrow \infty$ have been identified in [9]. For the high-$q^2$ region, potential normalizations are discussed in Appendix A, which could serve to form optimized observables for special scenarios of either vanishing chirality-flipped vector or tensor or scalar couplings. In the most general case, however, there are no optimized observables at high $q^2$. Although form factors do not cancel in this case, it might still be preferable to use normalizations, for example when the overall normalization of $B\rightarrow K^*$ form factors constitutes a major theoretical uncertainty.

**Table 1:** List of all observables of the various $b\rightarrow s \bar{\mu }\mu$ decays entering the fits with the respective kinematics and experiments that provide the measurements. LCSR and lattice results of $B\rightarrow K^{(*)}$ form factors are used to constrain a $q^2$-dependent form-factor parametrization. For more details see Sect. 3 and Appendix B

Channel	Constraints	Kinematics	Source
$B_s\rightarrow \bar{\mu }\mu$	$\overline{\mathscr {B}} \equiv \int \mathrm{d}\tau {\mathscr {B}}(\tau )$	–	[17–19]
${B^+\rightarrow K^+ \bar{\mu }\mu }$	${\mathscr {B}}_\mu$	$q^2\in [1,\, 6],\, [14.18,\, 16],\, [>\!\!16]$ GeV$^2$	[20]
		$q^2 \in [1.1,\, 6.0],\, [15.0,\, 22.0]$ GeV$^2$	[2]
	$A_\mathrm{FB}^\mu$	$q^2 \in [1.1,\, 6.0],\, [15.0,\, 22.0]$ GeV$^2$	[1, 20]
	$F_{H}^\mu$	$q^2 \in [1.1,\, 6.0],\, [15.0,\, 22.0]$ GeV$^2$	[1]
	$A_\mathrm{CP}^\mu$	$q^2 \in [1.1,\, 6.0],\, [15.0,\, 22.0]$ GeV$^2$	[3]
${B^0\rightarrow K^{*0} \bar{\mu }\mu }$	${\mathscr {B}}_\mu$	$q^2\in [1,\, 6],\, [14.18,\, 16],\, [>\!\!16]$ GeV$^2$	[20–22]
	$A_\mathrm{FB}^\mu$	$q^2\in [1,\, 6],\, [14.18,\, 16],\, [>\!\!16]$ GeV$^2$	[20–22]
	$A_\mathrm{CP}^\mu$	$q^2 \in [1.1,\, 6.0],\, [15.0,\, 22.0]$ GeV$^2$	[3]
${B\rightarrow K}$ form factors	$f_{0,+,T}$	$q^2 = 17,\, 20,\, 23$ GeV$^2$	[13]
${B\rightarrow K^*}$ form factors	$V,\,A_{0,1,2},\, T_{1,2,3}$	$q^2 = 0.1,\, 4.1,\, 8.1,\, 12.1$ GeV$^2$	[23]
	$V,\,A_{0,1,2},\, T_{1,2,3}$	$q^2 \in [11.9,\, 17.8]$ GeV$^2$	[24, 25]

To illustrate the sensitivity of $F_H^\ell$ to tensor couplings, we compare it in Fig. 1 to the branching ratios of $B\rightarrow K^{(*)} \bar{\ell }\ell$ for $\ell = \mu$, integrated over one low-$q^2$ and one high-$q^2$ bin. The details of the numerical input and the uncertainty propagation can be found in Appendices B and C. In the light of the hint of new physics in $\mathscr {C}_{9}^{\mathrm {}}$ from recent global analyses of $b\rightarrow s (\gamma ,\, \bar{\ell }\ell )$ data [26–30], we show predictions for $\mathscr {C}_{9}^{\mathrm {NP}} = -1.1$ in addition to $\mathscr {C}_{9}^{\mathrm {NP}} = 0$.

From Fig. 1, the highest sensitivity to tensor couplings of any $B \rightarrow K$ observable is attained by $F_H^\mu$ at high $q^2$ due to a partial cancellation of form factors [5]. If the experimental uncertainty could be reduced further, $F_H^\mu$ would give a very strong constraint on a simultaneous negative shift in $\mathscr {C}_{9}^{\mathrm {NP}}$ and $\mathscr {C}_{T}^{\mathrm {}}$. The prediction of ${\mathscr {B}}(B\rightarrow K \bar{\mu }\mu )$ is essentially insensitive to $\mathscr {C}_{T}^{\mathrm {}}$ but sensitive to $\mathscr {C}_{9}^{\mathrm {NP}}$. A stronger impact on global fits, however, would require a reduced theory uncertainty.

The observable ${\mathscr {B}}(B\rightarrow K^* \bar{\mu }\mu )$ shows moderate dependence on $\mathscr {C}_{T}^{\mathrm {}}$ at least at low $q^2$ and has some impact on the constraints on tensor couplings as will be discussed in Sect. 4. At the moment, theory and experimental uncertainty are of similar size.

$(J_{1c}+J_{2c})$ is sensitive to $\mathscr {C}_{T}^{\mathrm {}}$ in both $q^2$ regimes. At low $q^2$, it is mildly affected by $\mathscr {C}_{9}^{\mathrm {NP}}$, whereas at high $q^2$ it is unaffected. Regarding $(J_{1s}-3J_{2s})$, the situation is reversed: here the strong dependence on $\mathscr {C}_{T}^{\mathrm {}}$ appears at low $q^2$. Overall, $F_H^\mu$, $(J_{1c}+J_{2c})$ at high $q^2$, and $(J_{1s}-3J_{2s})$ are sensitive to $\mathscr {C}_{T}^{\mathrm {}}$ and theoretically very clean around $\mathscr {C}_{T}^{\mathrm {}}=0$.

From the available measurements, $F_H^\mu$ at high $q^2$ currently provides the most stringent constraints on the size of tensor couplings. Moreover, the dependence on vector couplings is such that $\mathscr {C}_{9}^{\mathrm {NP}} \lesssim 0$ leads to stronger constraints on $\mathscr {C}_{T}^{\mathrm {}}$ than $\mathscr {C}_{9}^{\mathrm {NP}} \approx 0$.

Important additional constraints on scalar couplings come from the branching ratio of $B_s \rightarrow \bar{\mu }\mu$ as given in (A.8). It provides the most stringent constraints on the moduli $|\mathscr {C}_{S}^{\mathrm {}} - \mathscr {C}_{S'}^{\mathrm {}}|$ and $|\mathscr {C}_{P}^{\mathrm {}} - \mathscr {C}_{P'}^{\mathrm {}}|$ and further depends only on $(\mathscr {C}_{10}^{\mathrm {}} - \mathscr {C}_{10'}^{\mathrm {}})$. Thus it is complementary to $F_H^{\ell }$ in $B\rightarrow K \bar{\ell }\ell$; see Eq. (3.6).

Eventually we also explore the effect of interference with NP contributions in the vector couplings $\mathscr {C}_{9,\,9',\,10,\,10'}^{\mathrm {}}$ on the bounds on tensor and scalar couplings. For this purpose we include also the branching ratio, the lepton forward–backward asymmetry, and the rate CP asymmetry of $B\rightarrow K^* \bar{\mu }\mu$ as they provide additional constraints on the real and imaginary parts of $\mathscr {C}_{9,\,9',\,10,\,10'}^{\mathrm {}}$. The experimental input of all observables entering our fits is listed in Table 1 together with input for the $B\rightarrow K^{(*)}$ form factors. More details on the latter can be found in Appendix B.

## Fits and constraints

There are no discrepancies between the latest measurements for $\ell = \mu$ (throughout this section) of $F_H^\mu$ and $A_\mathrm{FB}^\mu$ in $B^+ \rightarrow K^+ \bar{\mu }\mu$ and their tiny SM predictions; cf. Fig. 1. Thus our main objective is to derive constraints on tensor and scalar couplings through the enhanced sensitivity of both observables to these couplings compared to vector couplings. For this purpose, we will consider several model-independent scenarios, progressing from rather restricted to more general ones in order to asses the effect of cancellations due to interference of various contributions.

For each coupling that we vary in a fit, we remain as general as possible, treat it as a complex number and use the Cartesian parametrization assuming uniform priors for ease of comparison with previous studies. Specifically, we set

4.1 $$\begin{aligned} \text{ Re }(\mathscr {C}_{S,S',P,P',T,T5}^{\mathrm {}})&\in [-1, 1],\nonumber \\ \text{ Re }(\mathscr {C}_{9,9',10,10'}^{\mathrm {}})&\in [-7, 7], \end{aligned}$$

and the same for the imaginary parts. The priors of the nuisance parameters are given in Appendix B.

We start with the scenario of only tensor couplings and see that they are well constrained by $F_H^\mu$ alone. In a second scenario we consider only scalar couplings in order to investigate the complementarity of $F_H^\mu$ and $B_s \rightarrow \bar{\mu }\mu$. Here we find that – for the first time – all complex-valued scalar couplings can be bounded simultaneously by the combination of both measurements. Finally we consider as a special scenario the SM augmented by dimension-six operators as an effective theory of new physics below some high scale $\Lambda _\mathrm{NP}$ assumed much larger than the typical scale of electroweak symmetry breaking. In addition, the model contains one scalar doublet under $SU(2)_L$ as in the SM. For each scenario, we also investigate interference effects with new physics in vector couplings $\mathscr {C}_{9,9',10,10'}^{\mathrm {}}$. Finally, we conclude this section with posterior predictions – conditional on all experimental constraints – of the probable ranges of the not-yet-measured angular observables $J_{6c}$, $(J_{1c}+J_{2c})$ and $(J_{1s} - 3J_{2s}$) in $B\rightarrow K^*\bar{\mu }\mu$ and $A_{\Delta \Gamma }$ in $B_s\rightarrow \bar{\mu }\mu$.

**Table 2:** The constraints on complex-valued $\mathscr {C}_{T,\,T5}^{\mathrm {}}$ when using measurements of only $F_H^\mu$, $F_H^\mu$ and other data in Table 1 (except $\overline{\mathscr {B}}(B_s \rightarrow \bar{\mu }\mu )$), and finally allowing for complex-valued new-physics contributions to $\mathscr {C}_{9,\,10}^{\mathrm {}}$

Data set	Only $F_H^\mu$	$F_H^\mu$ + other	$F_H^\mu$ + other
Set of couplings	$\mathscr {C}_{T,\,T5}^{\mathrm {}}$	$\mathscr {C}_{T,\,T5}^{\mathrm {}}$	$\mathscr {C}_{T,\,T5,\, 9,\,10}^{\mathrm {}}$
Credibility level	68 %, 95 %	68 %, 95 %	68 %, 95 %
$\text{ Re }\,\mathscr {C}_{T}^{\mathrm {}}$	$[-0.32,\, 0.16]$, $[-0.52,\, 0.35]$	$[-0.29,\, 0.09]$, $[-0.43,\, 0.27]$	$[-0.23,\, 0.14]$, $[-0.39,\, 0.30]$
$\text{ Im }\,\mathscr {C}_{T}^{\mathrm {}}$	$[-0.25,\, 0.24]$, $[-0.44,\, 0.44]$	$[-0.19,\, 0.21]$, $[-0.37,\, 0.35]$	$[-0.17,\, 0.22]$, $[-0.33,\, 0.37]$
$\text{ Re }\,\mathscr {C}_{T5}^{\mathrm {}}$	$[-0.25,\, 0.24]$, $[-0.44,\, 0.44]$	$[-0.19,\, 0.17]$, $[-0.33,\, 0.33]$	$[-0.18,\, 0.16]$, $[-0.32,\, 0.32]$
$\text{ Im }\,\mathscr {C}_{T5}^{\mathrm {}}$	$[-0.24,\, 0.25]$, $[-0.44,\, 0.45]$	$[-0.21,\, 0.19]$, $[-0.37,\, 0.35]$	$[-0.20,\, 0.18]$, $[-0.36,\, 0.35]$

**Fig. 2:**
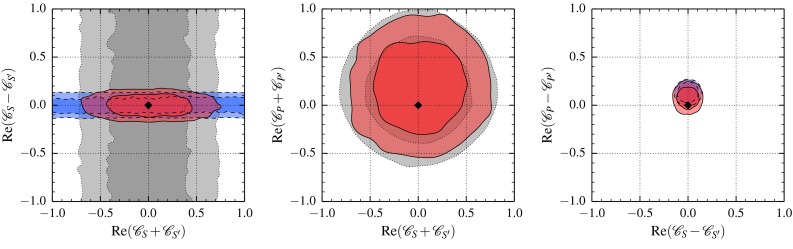
The constraints on complex-valued $\mathscr {C}_{S,\,S',\,P,\,P'}^{\mathrm {}}$ from only $F_H^\mu$ (*gray dotted*), only $\overline{\mathscr {B}}(B_s \rightarrow \bar{\mu }\mu )$ (*blue dashed*), and the combination with all other data in Table 1 as well as nonzero $\mathscr {C}_{9,\,9',\,10,\, 10'}^{\mathrm {}}$ (*red solid*) at 68 % (*darker*) and 95% (*lighter*) probability. The constraints on Re$(\mathscr {C}_{P}^{\mathrm {}} \pm \mathscr {C}_{P'}^{\mathrm {}})$ are identical to Re$(\mathscr {C}_{S}^{\mathrm {}} \pm \mathscr {C}_{S'}^{\mathrm {}})$, apart from a small translation of the contours by $(+0.2,\, +0.15)$. The SM prediction is indicated by the *black diamond*

### Tensor couplings

In a scenario with only complex-valued tensor couplings $\mathscr {C}_{T,\, T5}^{\mathrm {}}$, the experimental measurement of $F_H^\mu$ constrains the combination $|\mathscr {C}_{T}^{\mathrm {}}|^2 + |\mathscr {C}_{T5}^{\mathrm {}}|^2$, up to some small interference of $\mathscr {C}_{T}^{\mathrm {}}$ with vector couplings $\mathscr {C}_{7,\,7',\,9,\,9'}^{\mathrm {}}$; cf. (3.6). The according 68 % (95 %) 1D-marginalized probability intervals are listed in the second column of Table 2. From the third column, it is seen that the constraints become tighter when utilizing all observables in Table 1, mainly due to the sensitivity of the branching ratio of $B\rightarrow K^{*} \bar{\mu }\mu$ to tensor couplings (see also Fig. 1). The latter stronger bounds are driven by the new lattice results of $B\rightarrow K^*$ form factors that predict values above the measured ones [31]. Since tensor couplings contribute constructively to ${\mathscr {B}}(B\rightarrow K^{*}\bar{\mu }\mu )$, large values are better constrained. In this scenario with vanishing scalar couplings, current measurements of $A_\mathrm{FB}^\mu (B^+\rightarrow K^+\bar{\mu }\mu )$ barely provide any constraint; cf. (3.3).

We also perform a fit with nonzero $\mathscr {C}_{9,\,10}^{\mathrm {NP}}$ in order to assess the robustness of the bounds with respect to interference. Note that $\mathscr {C}_{7,7'}^{\mathrm {}}$ appears in linear combinations with $\mathscr {C}_{9,9'}^{\mathrm {}}$ such that its interference with tensor and scalar couplings is captured implicitly by allowing for new physics in $\mathscr {C}_{9,9'}^{\mathrm {}}$. Thus we fix $\mathscr {C}_{7,7'}^{\mathrm {}}$ to the SM value without loss of generality. In this case, $F_H^\mu$ by itself still provides bounds on $|\mathscr {C}_{T,\,T5}^{\mathrm {}}|$ that are weakened by a factor of 2, since $F_H^\mu$ does not pose constraints on $\mathscr {C}_{9,\,10}^{\mathrm {}}$ (in the chosen prior range). Once additional experimental measurements of Table 1 are taken into account, the potential destructive effects of new physics in $\mathscr {C}_{9,\,10}^{\mathrm {}}$ become reduced and almost the same constraints on $\mathscr {C}_{T,\,T5}^{\mathrm {}}$ are recovered, as shown in the last column in Table 2. If in addition we allow for $\mathscr {C}_{S,S',P,P'}^{\mathrm {}} \ne 0$ (not shown in Table 2), the credible regions further shrink by about 10%, which we attribute to the cumulative effect of $\mathscr {C}_{S,S',P,P'}^{\mathrm {}} \ne 0$ in $F_H^\ell$; cf. (3.6). In summary, the $F_H^\mu$ measurement [1] of LHCb with 3 fb$^{-1}$ shrinks the previous bounds [5] on $\mathscr {C}_{T,\,T5}^{\mathrm {}}$ by roughly 50 %.

**Table 3:** The 1D-marginalized constraints on complex-valued $\mathscr {C}_{S,\,S',\,P,\,P'}^{\mathrm {}}$ at 68% (95%) probability from measurements of only $F_H^\mu$, only $\overline{\mathscr {B}}(B_s \rightarrow \bar{\mu }\mu )$, and all the data in Table 1 and additional new-physics contributions to $\mathscr {C}_{9,\, 9',\, 10,\,10'}^{\mathrm {}}$

Data set	Only $F_H^\mu$	Only $\overline{\mathscr {B}}(B_s \rightarrow \bar{\mu }\mu )$	All
Set of couplings	$\mathscr {C}_{S,\,S',\,P,\,P'}^{\mathrm {}}$	$\mathscr {C}_{S,\,S',\,P,\,P'}^{\mathrm {}}$	$\mathscr {C}_{S,\,S',\,P,\,P',\,9,\,9',\,10,\,10'}^{\mathrm {}}$
Credibility level	68 %, 95 %	68 %, 95 %	68 %, 95 %
$\text{ Re }(\mathscr {C}_{S}^{\mathrm {}} - \mathscr {C}_{S'}^{\mathrm {}})$	–	$[-0.10,\, 0.08]$, $[-0.14,\, 0.13]$	$[-0.08,\, 0.07]$, $[-0.13,\, 0.13]$
$\text{ Im }(\mathscr {C}_{S}^{\mathrm {}} - \mathscr {C}_{S'}^{\mathrm {}})$	–	$[-0.07,\, 0.07]$, $[-0.11,\, 0.12]$	$[-0.07,\, 0.07]$, $[-0.12,\, 0.11]$
$\text{ Re }(\mathscr {C}_{S}^{\mathrm {}} + \mathscr {C}_{S'}^{\mathrm {}})$	$[-0.36,\, 0.39]$, $[-0.69,\, 0.68]$	$-$	$[-0.32,\, 0.32]$, $[-0.59,\, 0.62]$
$\text{ Im }(\mathscr {C}_{S}^{\mathrm {}} + \mathscr {C}_{S'}^{\mathrm {}})$	$[-0.37,\, 0.35]$, $[-0.68,\, 0.66]$	$-$	$[-0.25,\, 0.41]$, $[-0.57,\, 0.64]$
$\text{ Re }(\mathscr {C}_{P}^{\mathrm {}} - \mathscr {C}_{P'}^{\mathrm {}})$	$-$	$[ 0.05,\, 0.20]$, $[ 0.01,\, 0.26]$	$[ 0.00,\, 0.16]$, $[-0.07,\, 0.22]$
$\text{ Im }(\mathscr {C}_{P}^{\mathrm {}} - \mathscr {C}_{P'}^{\mathrm {}})$	$-$	$[-0.07,\, 0.08]$, $[-0.12,\, 0.12]$	$[-0.07,\, 0.09]$, $[-0.14,\, 0.16]$
$\text{ Re }(\mathscr {C}_{P}^{\mathrm {}} + \mathscr {C}_{P'}^{\mathrm {}})$	$[-0.24,\, 0.51]$, $[-0.51,\, 0.82]$	$-$	$[-0.12,\, 0.52]$, $[-0.42,\, 0.78]$
$\text{ Im }(\mathscr {C}_{P}^{\mathrm {}} + \mathscr {C}_{P'}^{\mathrm {}})$	$[-0.36,\, 0.37]$, $[-0.67,\, 0.67]$	$-$	$[-0.37,\, 0.29]$, $[-0.68,\, 0.57]$

### Scalar couplings

Scalar couplings $\mathscr {C}_{S,\, S',\, P,\, P'}^{\mathrm {}}$ enter $F_H^\ell$ without kinematic suppression – see (3.6) – as the sum $(\mathscr {C}_{i}^{\mathrm {}} + \mathscr {C}_{i'}^{\mathrm {}})$ whereas in the time-integrated branching ratio $\overline{\mathscr {B}}(B_s\rightarrow \bar{\ell }\ell )$ they appear as the difference $(\mathscr {C}_{i}^{\mathrm {}} - \mathscr {C}_{i'}^{\mathrm {}}), i = S,\, P$. Since the existing measurement of $F_H^\mu$ constrains the sum, the combination of $F_H^\mu$ and $\overline{\mathscr {B}}(B_s \rightarrow \bar{\mu }\mu )$ allows us – for the first time – to bound the real and imaginary parts of all four couplings. The corresponding 2D-marginalized regions in the $\text{ Re }(\mathscr {C}_{i}^{\mathrm {}} \pm \mathscr {C}_{i'}^{\mathrm {}})$ ($i = S, P$) planes are shown in Fig. 2. The corresponding plots for $\text{ Im }(\mathscr {C}_{i}^{\mathrm {}} \pm \mathscr {C}_{i'}^{\mathrm {}})$ are very similar to those shown and thus omitted. These bounds do not change when including all other data in Table 1, since $A_\mathrm{FB}^\mu (B\rightarrow K\bar{\mu }\mu )$ requires interference of scalar with tensor couplings and the other observables are not very sensitive to scalar couplings. Quantitatively, the constraint from $\overline{\mathscr {B}} (B_s \rightarrow \bar{\mu }\mu )$ on $(\mathscr {C}_{i}^{\mathrm {}} - \mathscr {C}_{i'}^{\mathrm {}})$ is about a factor 4 to 5 stronger than the one of $F_H^\mu$ on $(\mathscr {C}_{i}^{\mathrm {}} + \mathscr {C}_{i'}^{\mathrm {}})$.

Interference terms of $\mathscr {C}_{P,\,P'}^{\mathrm {}}$ with vector couplings might weaken these bounds. For $\overline{\mathscr {B}}(B_s \rightarrow \bar{\mu }\mu )$, the relevant term is $(\mathscr {C}_{10}^{\mathrm {}} - \mathscr {C}_{10'}^{\mathrm {}})$ (see (A.7)) and for $F_H^\ell$ it is $(\mathscr {C}_{10}^{\mathrm {}} + \mathscr {C}_{10'}^{\mathrm {}})$ [4]; both are suppressed by the factors $m_\mu /M_{B_s}$ and $m_\mu /\sqrt{q^2}$, respectively. Nevertheless, these terms become important for small $\mathscr {C}_{P,\,P'}^{\mathrm {}}$ due to the large SM value of $\mathscr {C}_{10}^{\mathrm {SM}} \simeq -4.2$. We compile bounds on complex-valued scalar couplings in Table 3 for only $F_H^\mu$, only $\overline{\mathscr {B}}(B_s \rightarrow \bar{\mu }\mu )$, and their combination with all the other observables in Table 1. Neither $F_H^\ell$ nor $\overline{\mathscr {B}}(B_s \rightarrow \bar{\ell }\ell )$ alone can bound all four complex-valued scalar couplings, however, their combination is capable to do so and moreover, the bounds are stable against destructive interference with vector couplings.

In the special case of real-valued couplings, $\overline{\mathscr {B}}(B_s \rightarrow \bar{\ell }\ell )$ would lead to rings [32] instead of circles in Fig. 2. Our results improve and extend previous bounds in the literature to the most general case of complex-valued couplings. For example they are a factor 2 to 5 more stringent than [33] and comparable to [34] once restricting to the simpler scenarios considered there.

### SM-EFT-constrained scalar couplings

In the following we consider a scenario in which it is assumed that there is a sizable hierarchy between the electroweak scale and the new-physics scale, $\Lambda _\mathrm{NP}$, and that the SM gauge symmetries $SU(2)_L \times U(1)_Y$ are only broken at the electroweak scale. This results in the augmentation of the SM by dimension-six operators that respect the SM gauge group and are composed of SM fields only. Such a scenario becomes more and more viable for two reasons. The first is the discovery of a scalar resonance at the LHC in agreement with all requirements of the Higgs particle in the SM. The second is the steadily rising lower bound on the mass of new particles reported by ATLAS and CMS in various more or less specific models.

A nonredundant set of dimension-six operators of this effective theory (SM-EFT) that requires a linear realization of the electroweak symmetry was given in [35]. The matching of the SM-EFT to the effective theory of $\Delta B = 1$ decays (2.1) at the scale $\mu \sim m_W$ of the order of the *W*-boson mass was performed for vector couplings $\mathscr {C}_{7,\,9,\,10}^{\mathrm {}}$ in [36]. The matching of tensor (2.4) and scalar (2.3) operators [32] shows that SM gauge groups in conjunction with the linear representation impose the relations

4.2 $$\begin{aligned} \mathscr {C}_{P}^{\mathrm {}}&= -\mathscr {C}_{S}^{\mathrm {}},&\mathscr {C}_{P'}^{\mathrm {}}&= \mathscr {C}_{S'}^{\mathrm {}},&\mathscr {C}_{T}^{\mathrm {}}&= \mathscr {C}_{T5}^{\mathrm {}} = 0 \end{aligned}$$

on scalar couplings and require tensor couplings to be suppressed to the level of dimension-eight operators. In consequence only two scalar couplings $\mathscr {C}_{S,\, S'}^{\mathrm {}}$ arise that scale as $({\sim }v/\Lambda _\mathrm{NP})^2 \ll 1$ where $v \propto m_W$ denotes the scale of electroweak symmetry breaking.

It must be noted that the relations (4.2) are a consequence of embedding the Higgs in a weak doublet along with the Goldstone bosons. For example, choosing a nonlinear representation of the scalar sector allows for additional dimension-six operators in the according effective theory, such that the couplings $\mathscr {C}_{S,S',P,P'}^{\mathrm {}}$ are all independent and tensor operators have nonvanishing couplings already at dimension six [37].

**Fig. 3:**
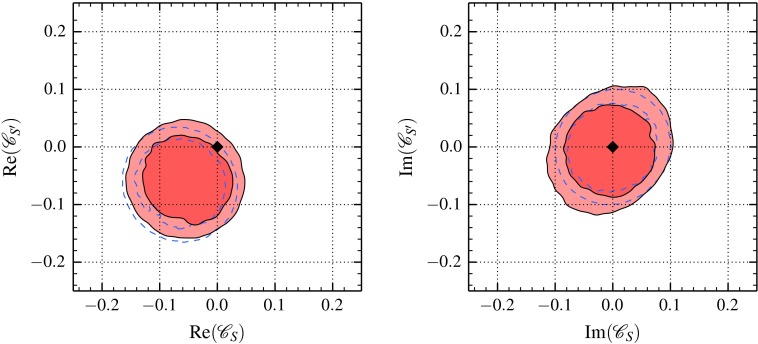
68 and 95 % contours of the 2D-marginalized distributions of scalar couplings $\mathscr {C}_{S,\,S'}^{\mathrm {}}$ in the scenario SM-EFT with all constraints in Table 1 (*red*) when marginalizing over nonzero $\text{ Re, } \text{ Im } (\mathscr {C}_{10,\,10'}^{\mathrm {NP}})$. For comparison, we superimpose the corresponding contours from using only $\overline{\mathscr {B}}(B_s\rightarrow \bar{\mu }\mu )$ and all $B \rightarrow K \bar{\mu }\mu$ constraints (*dashed blue contours*) with fixed $\mathscr {C}_{10,\,10'}^{\mathrm {NP}} = 0$. The SM is indicated by the *black diamond*

Omitting for the sake of simplicity terms of order $m_\ell ^2/M_{B_s}^2$ and $m_s/m_b$, the couplings $\mathscr {C}_{S,\, S'}^{\mathrm {}}$ can be bound from [32]4.3In a similar spirit, dropping terms of order $m_\ell ^2/q^2$ and $m_s/m_b$ gives

4.4 $$\begin{aligned} F_H^\ell&\propto \left| \mathscr {C}_{S}^{\mathrm {}} + \mathscr {C}_{S'}^{\mathrm {}}\right| ^2 + \left| \mathscr {C}_{S}^{\mathrm {}} - \mathscr {C}_{S'}^{\mathrm {}}\right| ^2\nonumber \\&\quad - \frac{4 m_\ell \, m_b}{q^2} \text{ Re } \left[ (\mathscr {C}_{S}^{\mathrm {}} - \mathscr {C}_{S'}^{\mathrm {}})(\mathscr {C}_{10}^{\mathrm {}} + \mathscr {C}_{10'}^{\mathrm {}})^* \right] . \end{aligned}$$

In the SM-EFT no relations between $\mathscr {C}_{10}^{\mathrm {}}$ and $\mathscr {C}_{10'}^{\mathrm {}}$ arise, so they are in general additional independent parameters. Here we find that destructive interference with contributions involving $\mathscr {C}_{10,\, 10'}^{\mathrm {}}$ does not significantly alter the bounds on $\mathscr {C}_{S,\, S'}^{\mathrm {}}$. The results of two fits are shown in Fig. 3. In the first fit, we set $\mathscr {C}_{10, 10'}^{\mathrm {NP}} = 0$ and include all constraints on $B \rightarrow K \bar{\mu }\mu$ and $B_s \rightarrow \bar{\mu }\mu$. In the second fit, we allow for $\mathscr {C}_{10, 10'}^{\mathrm {NP}} \ne 0$ and further include all $B \rightarrow K^* \bar{\mu }\mu$ constraints from Table 1. For both fits, all six 2D marginals of real and imaginary parts of $\mathscr {C}_{S}^{\mathrm {}}$ vs. $\mathscr {C}_{S'}^{\mathrm {}}$ have nearly circular contours of equal size that contain the SM point at the 68 % level except for $\text{ Re }(\mathscr {C}_{S}^{\mathrm {}})$ vs. $\text{ Re }(\mathscr {C}_{S'}^{\mathrm {}})$ where it is within the 95 % credible region. The regions hardly vary between the two fits.

Since we consider here complex-valued couplings the allowed regions are circles rather than rings as for the case of real-valued couplings [32]. Compared to those rings, the circles are smaller because the probability moves from the ring toward the center of the circle.

### Tensor, scalar, and vector couplings 

The most general fit of complex-valued tensor and scalar couplings $\mathscr {C}_{S,S',P,P',T,T5}^{\mathrm {}}$ in combination with vector couplings $\mathscr {C}_{9,\,9',\,10,\, 10'}^{\mathrm {}}$ – the combination of Sect. 4.1 and Sect. 4.2 – yields bounds very similar to those in Tables 2 and 3. The changes are only small and in fact the bounds tend to be even more stringent by $\sim$(10–20) % because tensor and scalar couplings can contribute to $F_H^{\mu }$ only constructively – see (3.6). As before, branching-ratio measurements of $B\rightarrow K^{*}\bar{\mu }\mu$ help to improve the constraints on $\mathscr {C}_{T,\, T5}^{\mathrm {}}$. This demonstrates that even in the case of complex-valued couplings there is enough information in the data to bound all 20 real and imaginary parts.

**Table 4:** The posterior predictive 68 % probability intervals of not-yet-measured angular observables for several new-physics scenarios given all the considered experimental constraints. The corresponding values for the SM (prior predictive) are given, too, where “$\simeq 0$” indicates zero in the considered approximation, see text for details

Observable	$q^2$-bin [GeV$^2$]	SM	$T(5),\,9,\,10$	$S^{(')},\,P^{(')},\,9^{(')},\,10^{(')}$	$S^{(')},P^{(')},\,T(5),\,9^{(')},10^{(')}$
$\tau _{B^0} \times J_{6c}$	$[1.1,\, 6]$	${\simeq }$0	$(0.6_{-1.9}^{+1.8}) \times 10^{-9\;}$	$(-0.1_{-1.9}^{+2.3}) \times 10^{-10}$	$(0.2_{-1.1}^{+1.1}) \times 10^{-9}$
	$[15,\, 19]$	${\simeq }0$	$(2.1_{-6.2}^{+5.1}) \times 10^{-10}$	$(0.7_{-6.6}^{+5.4}) \times 10^{-11}$	$(-0.2_{-2.9}^{+3.5}) \times 10^{-11}$
$\tau _{B^0} \times (J_{1c} + J_{2c})$	$[1.1,\, 6]$	$(3.30_{-0.56}^{+0.65}) \times 10^{-9\;}$	$(4.8_{-1.9}^{+1.7}) \times 10^{-9\;}$	$(2.6_{-0.4}^{+0.5}) \times 10^{-9\;}$	$(2.8_{-1.1}^{+1.5}) \times 10^{-9}$
	$[15,\, 19]$	$(1.72_{-0.16}^{+0.16}) \times 10^{-10}$	$(4.7_{-2.6}^{+3.3}) \times 10^{-9\;}$	$(1.9_{-0.7}^{+0.3}) \times 10^{-10}$	$(3.1_{-2.3}^{+2.5}) \times 10^{-9}$
$\tau _{B^0} \times (J_{1s} - 3 J_{2s})$	$[1.1,\, 6]$	$(2.44_{-0.48}^{+0.46}) \times 10^{-10}$	$(1.9_{-1.1}^{+1.4}) \times 10^{-8\;}$	$(4.0_{-1.2}^{+1.6}) \times 10^{-10}$	$(1.5_{-1.1}^{+1.0}) \times 10^{-8}$
	$[15,\, 19]$	$(1.12_{-0.10}^{+0.10}) \times 10^{-10}$	$(7.1_{-3.8}^{+5.6}) \times 10^{-9\;}$	$(1.4_{-0.4}^{+0.3}) \times 10^{-10}$	$(5.3_{-3.8}^{+3.8}) \times 10^{-9}$
$A_{\Delta \Gamma }(B_s \rightarrow \bar{\mu }\mu )$	–	1	1	$[-1,\, 1]$	$[-1,\, 1]$

### Angular observables in $B\rightarrow K^* \bar{\ell }\ell$

Now we discuss what the fits tell us about likely values of observables that have not been measured yet but have sensitivity to tensor and scalar couplings. In the $B\rightarrow K^*(\rightarrow K\pi )\bar{\ell }\ell$ decay, we again consider $(J_{1s} - 3\, J_{2s})$ and $(J_{1c} + J_{2c})$ as in Sect. 3 and additionally $J_{6c}$. We compute the posterior predictive distribution (see Appendix C) for each observable integrated over the low-$q^2$ bin $[1.1,\, 6]$ GeV$^2$ and high-$q^2$ bin $[15,\, 19]$ GeV$^2$ matching LHCb’s range. The distributions resemble Gaussians, thus we summarize them by their modes and smallest 68 % intervals in Table 4 comparing the SM (prior predictive, $\mathscr {C}_{i}^{\mathrm {NP}}=0$) to three NP scenarios. In each, we allow for interference with the vector couplings and additionally vary only $\mathscr {C}_{T,T5}^{\mathrm {}}$ (Sect. 4.1), only $\mathscr {C}_{S,S',P,P'}^{\mathrm {}}$ (Sect. 4.2), and finally both tensor and scalar couplings (Sect. 4.4).

We rescale $J_i$ and combinations by the $B^0$-meson life time $\tau _{B^0} = 1.519$ ps [38] to judge the experimental sensitivity in the near future by comparing to current measurements of the branching ratio,

4.5 $$\begin{aligned} {{\mathscr {B}}}&= \frac{\tau _{B^0}}{3} \left[ 2\, (3 J_{1s} - J_{2s}) + (3 J_{1c} - J_{2c})\right] . \end{aligned}$$

In the SM, the typical magnitude of the branching ratio of $B\rightarrow K^*\bar{\ell }\ell$ is approximately equal to $2 \times 10^{-7}$ for both the $q^2 \in [1.1,\, 6]$ and the $[15,\, 19]$ GeV$^2$ bins. For comparison, the predicted ranges for $\tau _{B^0}(J_{1s} - 3\, J_{2s})$ and $\tau _{B^0}(J_{1c} + J_{2c})$ in the SM are suppressed by $2-3$ orders of magnitude down to ${\mathscr {O}}(10^{-10})$; cf. Table 4.

The angular observable $J_{6c}$ is strictly zero in the absence of tensor and scalar couplings. Nonzero contributions can be generated in the SM by QED corrections or potentially from higher-dimensional ($d \ge 8$) $|\Delta B| = |\Delta S| = 1$ operators, leading to parametric suppression by $\alpha _e/(4 \pi )$ or $m_b m_\ell /m_W^2$. These factors should be compared to the potential suppression present for tensor and scalar contributions in particular NP models in order to gauge their relevance. Our model-independent fits are still in a regime where such considerations are insignificant since current experimental measurements, in combination with theory uncertainties, do not yet impose sufficiently stringent constraints on tensor and scalar couplings.

Beyond the SM, $J_{6c}$ can become of order ${\mathscr {O}}(10^{-9})$ in scenarios involving tensor couplings only and about ${\mathscr {O}}(10^{-10})$ in the presence of scalar couplings only. Both effects are due to interference with vector couplings. Schematically, $J_{6c}$ is a function of $\mathscr {C}_{T}^{\mathrm {}} \times \mathscr {C}_{P}^{\mathrm {}} + \mathscr {C}_{S}^{\mathrm {}} \times \mathscr {C}_{T5}^{\mathrm {}}$, $m_{\ell }/\sqrt{q^2} \times \text{ scalar } \times \text{ vector }$, and $m_{\ell }/\sqrt{q^2} \times \text{ tensor } \times \text{ vector }$. The largest interval is obtained for the scenario without scalar couplings because then the uncertainty on the (tensor) couplings is largest. But even then, it seems that the experimental sensitivity will not be high enough to have an impact in global fits.

Concerning $(J_{1c} + J_{2c})$ and $(J_{1s} - 3\, J_{2s})$, substantial deviations from the SM prediction are again only possible in the presence of nonzero tensor couplings. In this case, an enhancement by two orders of magnitude is possible up to ${\mathscr {O}}(10^{-8})$ at high $q^2$ and also at low $q^2$ in the case of $(J_{1s} - 3\, J_{2s})$. We want to stress again that we make these statements conditional on all included experimental constraints, the scenario, and our prior. In view of the current experimental precision of $20\%$ on the branching ratio at LHCb [21] with only 1 fb$^{-1}$, corresponding to the ${\mathscr {O}}(10^{-8})$, one can indeed hope for some sensitivity to such large effects in $(J_{1c} + J_{2c})$ and $(J_{1s} - 3\, J_{2s})$ for the not-yet-published 3 fb$^{-1}$ data set. At least, we can hope for some measurement if the method of moments [7] is applied.

For the mass-eigenstate rate asymmetry $A_{\Delta \Gamma }(B_s\rightarrow \bar{\mu }\mu )$ induced by the nonvanishing width of the $B_s$ meson (cf. Appendix A), we find a rather uniform distribution in scenarios with nonzero scalar couplings. So any value in the range $[-1,\, 1]$ is plausible whereas the SM and the scenario with only tensor couplings predict a value of precisely one [39]; cf. the last row in Table 4. Hence any deviation from one would unambiguously hint at the presence of scalar operators.

## Conclusions

We have derived the most stringent constraints to date on tensor and scalar couplings that mediate $b\rightarrow s\bar{\mu }\mu$ transitions. They are based on the latest measurements of angular observables $F_H^\mu$ and the lepton forward–backward asymmetry $A_\mathrm{FB}^\mu$ in $B^+\rightarrow K^+ \bar{\mu }\mu$ from LHCb [1], supplemented by measurements of the branching ratios of $B_s\rightarrow \bar{\mu }\mu$ and $B\rightarrow K^{(*)} \bar{\mu }\mu$.

Both $F_H^\mu$ and $A_\mathrm{FB}^\mu$ belong to a class of observables in which vector and dipole couplings – present in the standard model (SM) – are suppressed (mostly kinematically by $m_\ell / \sqrt{q^2}$) with respect to tensor and scalar couplings. We provide predictions for the equivalent but not-yet-measured angular observables $J_{6c}$, $(J_{1c} + J_{2c})$, and $(J_{1s} - 3 J_{2s})$ in $B\rightarrow K^* (\rightarrow K\pi ) \bar{\mu }\mu$.

In a Bayesian analysis of the complex-valued couplings of the effective theory, we find that the measurement of $F_H^\mu$, especially at high-$q^2$,

1. imposes by itself constraints on real and imaginary parts of the tensor couplings $\mathscr {C}_{T,\,T5}^{\mathrm {}}$ such that the upper bound of the smallest 68 % (95 %) credibility interval is $\lesssim 0.25\, (0.45)$, superseding previous bounds. In combination with current data from $B\rightarrow K^* \bar{\mu }\mu$ and lattice predictions of $B\rightarrow K^*$ form factors, the bounds are lowered to $\lesssim 0.23\, (0.39)$, even in the presence of nonstandard contributions in vector couplings.
2. for the first time allows one to simultaneously bound all four scalar couplings $\mathscr {C}_{S,S',P,P'}^{\mathrm {}}$ due to its complementarity to $\overline{\mathscr {B}}(B_s \rightarrow \bar{\mu }\mu )$. Even when taking into account destructive interference with vector couplings, all credibility intervals contain the SM. The corresponding 1D intervals of real and imaginary parts have widths of $\sim$ 0.6 (1.2) for $\mathscr {C}_{i}^{\mathrm {}} + \mathscr {C}_{i'}^{\mathrm {}}$ and $\sim$0.15  (0.3) for $\mathscr {C}_{i}^{\mathrm {}} - \mathscr {C}_{i'}^{\mathrm {}}$ for $i = S,P$. Currently, the bounds from $F_H^\mu$ are weaker than those from $\overline{\mathscr {B}}(B_s \rightarrow \bar{\mu }\mu )$ by about a factor of 4. Future measurements of $F_H^\mu$ at LHCb and Belle II will further tighten the bounds. Moreover, measurements of $F_H^e$ ($\ell = e$) will provide constraints on scalar couplings in the electron channel in the absence of a direct determination of the branching ratio of $B_s \rightarrow \bar{e}e$.

Our updated bounds on complex-valued tensor and scalar couplings are summarized in Tables 2 and 3, accounting also for interference effects with vector couplings. These bounds hold even in the most general scenario of complex-valued tensor, scalar, and vector couplings, showing that the data are good enough to bound the real and imaginary parts of all Wilson coefficients simultaneously.

As a special case, we consider the scenario arising from the SM augmented by dimension-six operators generalizing existing studies to the case of complex-valued couplings. In this scenario, tensor couplings are absent and additional relations between scalar couplings are enforced by the linear realization of the $SU(2)_L \otimes U(1)_Y$ electroweak symmetry group.

Our study of the yet unmeasured angular observables $J_{6c}$, $(J_{1c} + J_{2c})$, and $(J_{1s} - 3 J_{2s})$ in $B\rightarrow K^* (\rightarrow K\pi ) \bar{\mu }\mu$ (see Table 4) shows that despite the current bounds on tensor couplings, enhancements of up to two orders of magnitude over the SM predictions are allowed for $(J_{1c} + J_{2c})$ and $(J_{1s} - 3 J_{2s})$, placing them in the reach of the LHCb analysis of the full run I data set. Our bounds on scalar couplings from $B_s\rightarrow \bar{\mu }\mu$ and $F_H^\mu$, however, are already quite restrictive, permitting only small deviations from SM predictions in $(J_{1c} + J_{2c})$ and $(J_{1s} - 3 J_{2s})$. Notably, the mass-eigenstate rate asymmetry $A_{\Delta \Gamma }(B_s\rightarrow \bar{\mu }\mu )$ given nonzero scalar couplings can take on any value in the range $[-1,\, 1]$.

## Appendix A: Angular observables in $B\rightarrow K^* \bar{\ell }\ell$

Here we focus on those angular observables $J_i$ in $B\rightarrow K^* \bar{\ell }\ell$ in which tensor and scalar contributions are kinematically enhanced by a factor $\sqrt{q^2}/m_\ell$ compared to the vector contributions of the SM or the respective interference terms. The results and the notation follow [5].

For convenience, we split the time-like transversity amplitude $A_t$ into two parts as

A.1 $$\begin{aligned} A_t&= \tilde{A}_t - \frac{1}{2} \frac{\sqrt{q^2}}{m_\ell } A_P \, . \end{aligned}$$

Both terms depend on the scalar $B\rightarrow K^*$ form factor $A_0(q^2)$, a normalization factor *N*, and the Källén-function $\lambda$ (see [5]), but they have a different dependence on the Wilson coefficients $i = 10, 10', P, P'$:

A.2 $$\begin{aligned} \tilde{A}_t&= 2 N \frac{\sqrt{\lambda }}{\sqrt{q^2}} (\mathscr {C}_{10}^{\mathrm {}} - \mathscr {C}_{10'}^{\mathrm {}}) A_0,\nonumber \\ A_P&= - 2 N\sqrt{\lambda } \frac{(\mathscr {C}_{P}^{\mathrm {}} - \mathscr {C}_{P'}^{\mathrm {}})}{(m_b+m_s)} A_0. \end{aligned}$$

The lepton-flavor index $\ell$ of Wilson coefficients is omitted for brevity throughout.

In full generality, the angular observables in $B\rightarrow K^* \bar{\ell }\ell$ depend on seven transversity amplitudes with vector and dipole contributions, $A_{0,\,\perp ,\,\parallel }^{L,R}$ and $\tilde{A}_t$, one scalar and one pseudoscalar amplitude, $A_{S,\,P} \propto (\mathscr {C}_{S,P}^{\mathrm {}} - \mathscr {C}_{S',P'}^{\mathrm {}})$, and six tensor amplitudes, $A_{\parallel \perp ,\,t\perp ,\,0\parallel } \propto \mathscr {C}_{T}^{\mathrm {}}$ and $A_{t0,\,t\parallel ,\,0\perp } \propto \mathscr {C}_{T5}^{\mathrm {}}$. The interesting combinations are

A.3 $$\begin{aligned} \frac{4}{3} J_{6c}&= 4\, \beta _\ell \, \text{ Re } \left[ 2\, (A_{t0}^{} A_S^* - A_{\parallel \perp }^{} A_P^*)\right. \nonumber \\&\quad \left. + \frac{m_\ell }{\sqrt{q^2}} \left[ (A_0^L + A_0^R) A_S^* + 4\, A_{\parallel \perp }^{} \tilde{A}_t^* \right] \right] , \end{aligned}$$

A.4 $$\begin{aligned} \frac{4}{3} \left( J_{1c} + J_{2c}\right)&= \left| \frac{2\, m_\ell }{\sqrt{q^2}} (A_0^L + A_0^R) + 4\, A_{t0} \right| ^2\nonumber \\&\quad + 16\, \beta _\ell ^2\, |A_{\parallel \perp }|^2 \nonumber \\&\quad + \left| \frac{2\, m_\ell }{\sqrt{q^2}}\, \tilde{A}_t - A_P \right| ^2 + \beta _\ell ^2\, |A_S|^2, \end{aligned}$$

A.5 $$\begin{aligned} \frac{4}{3} \left( J_{1s} - 3\, J_{2s} \right)&= \left| \frac{\sqrt{2}\, m_\ell }{\sqrt{q^2}} (A_\perp ^L + A_\perp ^R) + 4\, A_{t\perp } \right| ^2 \nonumber \\&\quad + \left| \frac{\sqrt{2}\, m_\ell }{\sqrt{q^2}} (A_\parallel ^L + A_\parallel ^R) + 4\, A_{t\parallel }\right| ^2. \end{aligned}$$

The function $\beta _\ell ^2(q^2) \equiv 1 - 4 m_\ell ^2/q^2$ tends to 1 for $m_\ell \ll \sqrt{q^2}$. This condition is well fulfilled for $\ell = e$ and $q^2 \gtrsim 1\, \text{ GeV }^2$, provided that the tensor and scalar Wilson coefficients do not receive additional suppression factors. For $\ell = \mu$, the value of $q^2$ should not be too low, whereas in the case $\ell = \tau$, these observables are not anymore dominated by tensor and scalar contributions alone, and the full lepton-mass dependence has to be taken into account. Finally, we note that the second part of $(J_{1c} + J_{2c})$ in (A.4),

A.6 $$\begin{aligned}&\left| \frac{2\, m_\ell }{\sqrt{q^2}}\, \tilde{A}_t - A_P \right| ^2 + \beta _\ell ^2\, |A_S|^2 = 4 N^2 \lambda A_0^2 \left\{ \beta _\ell ^2 \left| \frac{\mathscr {C}_{S}^{\mathrm {}} - \mathscr {C}_{S}^{\mathrm '}}{m_b + m_s}\right| ^2\nonumber \right. \\&\quad \left. + \left| \frac{\mathscr {C}_{P}^{\mathrm {}} - \mathscr {C}_{P}^{\mathrm '}}{m_b + m_s} + \frac{2 m_\ell }{q^2} \left( \mathscr {C}_{10}^{\mathrm {}} - \mathscr {C}_{10}^{\mathrm '}\right) \right| ^2 \right\} , \end{aligned}$$

resembles very much the branching ratio of the rare decay $B_s \rightarrow \bar{\ell }\ell$ in the limit $q^2 \rightarrow M_{B_s}^2$

A.7 $$\begin{aligned} {\mathscr {B}}(B_s \rightarrow \bar{\ell }\ell )&= \frac{G_F^2 \alpha _e^2 |V_{tb}^{} V_{ts}^*|^2}{64\, \pi ^3} M_{B_s}^5 f_{B_s}^2 \tau _{B_s} \beta _\ell (q^2 = M_{B_s}^2) \nonumber \\&\quad \times \left\{ \beta _\ell ^2(q^2 = M_{B_s}^2) \left| \frac{\mathscr {C}_{S}^{\mathrm {}} - \mathscr {C}_{S}^{\mathrm '}}{m_b + m_s}\right| ^2 \right. \nonumber \\&\quad \left. + \left| \frac{\mathscr {C}_{P}^{\mathrm {}} - \mathscr {C}_{P}^{\mathrm '}}{m_b + m_s} + \frac{2 m_\ell }{M_{B_s}^2} \left( \mathscr {C}_{10}^{\mathrm {}} - \mathscr {C}_{10}^{\mathrm '}\right) \right| ^2 \right\} . \end{aligned}$$

Hence there is some similarity between $(J_{1c} + J_{2c})$ in $B \rightarrow K^* \bar{\ell }\ell$ and ${\mathscr {B}}(B_s \rightarrow \bar{\ell }\ell )$ in their dependence on the couplings but the former has additional dependence on tensor and vector couplings through the other transversity amplitudes. In $B \rightarrow K^* \bar{\ell }\ell$, the helicity suppression factor $4\,m_\ell ^2/q^2$ of vector couplings is weaker than the corresponding factor $4\,m_\ell ^2/M_{B_s}^2$ in $B_s \rightarrow \bar{\ell }\ell$.

Concerning $B_s\rightarrow \bar{\ell }\ell$, the expression (A.7) corresponds to the plain branching ratio at time $t=0$. Due to the nonvanishing decay width $\Delta \Gamma _s$, experiments measure the average time-integrated branching ratio – denoted by $\overline{\mathscr {B}}$ – and the two are related as [39]

A.8

Here $y_s \equiv \Delta \Gamma _s/(2\Gamma _s)$ with the numerical value given in [28]. $A_{\Delta \Gamma }$ is the mass-eigenstate rate asymmetry due to the nonvanishing width difference, which is $A_{\Delta \Gamma } = 1$ in the SM, but in general it can be $A_{\Delta \Gamma } \in [-1,\, 1]$. Since $A_{\Delta \Gamma }$ can depart from its SM value in the scenarios of new physics considered in this work, we take this effect into account in our numerical analysis, although it is suppressed by small $y_s$. The latest SM prediction $\overline{\mathscr {B}} (B_s \rightarrow \bar{\mu }\mu ) = (3.65 \pm 0.23) \times 10^{-9}$ [41] includes NLO electroweak [42] and NNLO QCD corrections [43].

Finally, we discuss the possibility of suitable normalizations of $J_{6c}$, $(J_{1s} - 3\, J_{2s})$, and $(J_{1c} + J_{2c})$ at high $q^2$ that would provide optimized observables. For this purpose we use form-factor relations at leading order in $1/m_b$ and neglect terms suppressed by $m_\ell /\sqrt{q^2}$. With the notation and expressions derived in [5],

A.9 $$\begin{aligned} J_{1s} - 3\, J_{2s}&= \frac{3}{2}\, \rho _1^T (f_\perp ^2 + f_\parallel ^2),\nonumber \\ J_{1c} + J_{2c}&= 3\, \rho _1^T f_0^2 + \frac{3 N^2 \lambda }{(m_b+m_s)^2} A_0^2 \nonumber \\&\quad \times \left( |\mathscr {C}_{S}^{\mathrm {}}-\mathscr {C}_{S'}^{\mathrm {}}|^2 + |\mathscr {C}_{P}^{\mathrm {}}-\mathscr {C}_{P'}^{\mathrm {}}|^2\right) . \end{aligned}$$

Here $f_{\perp , \parallel , 0}$ and $A_0$ denote $B\rightarrow K^*$ form factors, whereas the $\rho _1^\pm$ depends on vector couplings and $\rho _1^T \propto (|\mathscr {C}_{T}^{\mathrm {}}|^2 + |\mathscr {C}_{T5}^{\mathrm {}}|^2)$.

Concerning $(J_{1s} - 3\, J_{2s})$, there are no appropriate normalizations, unless the chirality-flipped $\mathscr {C}_{7',\,9',\,10'}^{\mathrm {NP}} = 0$ because then $\rho _1^+ = \rho _1^-$ holds. There are three potential normalizations

A.10 $$\begin{aligned}&\frac{4}{3} J_{1s} = \left( 3 \rho _1^+ + \rho _1^T \right) f_\perp ^2 + \left( 3 \rho _1^- + \rho _1^T\right) f_\parallel ^2, \nonumber \\&\frac{8}{3} J_{2s} = \left( \rho _1^+ - \rho _1^\mathrm{T}\right) f_\perp ^2 + \left( \rho _1^- - \rho _1^\mathrm{T}\right) f_\parallel ^2, \nonumber \\&J_{1s} + 2 J_{2s} = 3 \left( \rho _1^+ f_\perp ^2 + \rho _1^- f_\parallel ^2\right) \end{aligned}$$

where the last one depends only on vector couplings; i.e., is free of tensor and scalar ones. In $J_{1s}$ and $J_{2s}$, tensor couplings contribute either cumulatively or destructively to vector couplings in $\rho _1^\pm$.

A similar situation arises for $(J_{1c} + J_{2c})$, where no appropriate normalization exists, unless scalar couplings vanish. In this special case, both $J_{1c}$ and $J_{2c} = -3/2 (\rho _1^- - \rho _1^T) f_0^2$ depend only on $f_0$ and can be used as normalization. In the general case, $J_{2c}$ still depends only on tensor couplings and was used at low $q^2$ [9]. Finally we note that

A.11 $$\begin{aligned} J_{1c} - J_{2c}&= 3\,\rho _1^- f_0^2 + \frac{3 N^2 \lambda }{(m_b+m_s)^2} A_0^2 \nonumber \\&\quad \times \left( |\mathscr {C}_{S}^{\mathrm {}}-\mathscr {C}_{S'}^{\mathrm {}}|^2 + |\mathscr {C}_{P}^{\mathrm {}}-\mathscr {C}_{P'}^{\mathrm {}}|^2\right) . \end{aligned}$$

is free of tensor couplings at high $q^2$ and would provide access to $\rho _1^-$ provided scalar couplings vanish.

## Appendix B: Theoretical inputs

Here we describe the theoretical treatment of observables and collect the numerical input for the relevant parameters. The software package EOS [12, 40, 44] is used for the calculation of observables in $B_s\rightarrow \bar{\mu }\mu$ and $B\rightarrow K^{(*)}\bar{\ell }\ell$ and associated constraints. Both the likelihood and the prior are defined entirely within EOS.

Concerning the numerical input, we refer the reader to [28] for the compilation of nuisance parameters relevant to this work. We adopt the same values for fixed parameters and the same priors unless noted otherwise below. Specifically, we use identical priors for common nuisance parameters of the CKM quark-mixing matrix, the charm and bottom quark masses in the $\overline{\mathrm{MS}}$ scheme, and the parametrization of subleading corrections in $1/m_b$ as given in [28].

Contrary to [28], we do not choose a log-gamma distribution for the asymmetric uncertainties in priors anymore but rather a continuous yet asymmetric Gaussian distribution. This avoids a poor fit because the log-gamma distribution falls off too rapidly in the “short” tail. In a unifying spirit, we now use the continuous rather than discontinuous asymmetric Gaussian to approximate asymmetric experimental intervals in our likelihood.

The updated prior of the $B_s$ decay constant $f_{B_s}$ enters the branching ratio of $B_s\rightarrow \bar{\mu }\mu$. We adopt recent updates of the $N_f = (2 + 1)$ FLAG compilation [45]; see Table 5, which averages the results of [46–48]. More recent calculations with $N_f = (2+1+1)$ [49] and $N_f = 2$ [50] flavors are consistent with these averages.

The tensor and scalar amplitudes in $B\rightarrow K^{(*)} \bar{\ell }\ell$ factorize naively, i.e. they depend only on scalar and tensor $B\rightarrow K^{(*)}$ form factors. These amplitudes are implemented in EOS for $B\rightarrow K \bar{\ell }\ell$ and $B\rightarrow K^* \bar{\ell }\ell$ as given in [4] and [5], respectively and we refrain from using form-factor relations at low and high $q^2$ for the tensor and scalar $B\rightarrow K$ and $B\rightarrow K^*$ form factors.

Therefore, we need additional nuisance parameters for the complete set of $B\rightarrow K$ form factors $f_{+,T,0}$. As a consequence of using the *z* parametrization [51] and the kinematic relation $f_0(0) = f_+(0)$, five nuisance parameters (listed in Table 5) are needed. As prior information, we average the LCSR results [51] and [52] (see Table 5 and cf. [28]) supplement them with lattice determinations [13]. The lattice results are given in a slightly different parametrization necessitating a conversion to the parametrization used here. For this purpose, we generate the form factors from the parametrization of [13], including full correlations, at three values of $q^2 = 17,\, 20,\, 23$ GeV$^2$. Subsequently, these “data” are included in the prior by means of a multivariate Gaussian whose mean and covariance is given in Table 6. The $q^2$ values and number of points are chosen such that the correlation of neighboring points is small enough to keep the covariance nonsingular.

**Table 5:** Prior distributions of the nuisance parameters for hadronic quantities

Quantity	Prior	References
$B_s$ decay constant		
$f_{B_s}$	($227.7 \pm 4.5$) MeV	[45]
$B\rightarrow K$ form factors		
$f_+(0)=f_0(0)$	$0.34 \pm 0.05$	[51, 52]
$f_T(0)$	$0.38 \pm 0.06$	[51, 52]
$b_1^0$	$-4.3^{+0.8}_{-0.9}$	[51]
$b_1^+$	$-2.1^{+0.9}_{-1.6}$	[51]
$b_1^T$	$-2.2^{+1.0}_{-2.0}$	[51]
$B\rightarrow K^*$ form factors		
$\alpha _{0}^{A_0}$	$0.35^{+0.02}_{-0.03}$	[23, 25]
$\alpha _{1}^{A_0}$	$-1.21^{+0.08}_{-0.08}$	[23, 25]
$\alpha _{2}^{A_0}$	$0.77^{+0.45}_{-0.48}$	[23, 25]
$\alpha _{0}^{A_1}$	$0.2625^{+0.015}_{-0.015}$	[23, 25]
$\alpha _{1}^{A_1}$	$0.07^{+0.08}_{-0.08}$	[23, 25]
$\alpha _{2}^{A_1}$	$0.045^{+0.12}_{-0.15}$	[23, 25]
$\alpha _{1}^{A_{12}}$	$0.53^{+0.10}_{-0.14}$	[23, 25]
$\alpha _{2}^{A_{12}}$	$0.32^{+0.33}_{-0.39}$	[23, 25]
$\alpha _{0}^{V}$	$0.35^{+0.02}_{-0.03}$	[23, 25]
$\alpha _{1}^{V}$	$-1.19^{+0.08}_{-0.08}$	[23, 25]
$\alpha _{2}^{V}$	$1.55^{+0.33}_{-0.33}$	[23, 25]
$\alpha _{0}^{T_1}$	$0.31^{+0.02}_{-0.02}$	[23, 25]
$\alpha _{1}^{T_1}$	$-1.07^{+0.08}_{-0.08}$	[23, 25]
$\alpha _{2}^{T_1}$	$1.40^{+0.30}_{-0.30}$	[23, 25]
$\alpha _{1}^{T_2}$	$0.33^{+0.08}_{-0.10}$	[23, 25]
$\alpha _{2}^{T_2}$	$0.26^{+0.21}_{-0.24}$	[23, 25]
$\alpha _{0}^{T_{23}}$	$0.67^{+0.04}_{-0.05}$	[23, 25]
$\alpha _{1}^{T_{23}}$	$0.93^{+0.28}_{-0.22}$	[23, 25]
$\alpha _{2}^{T_{23}}$	$-0.15^{+0.86}_{-0.71}$	[23, 25]

**Table 6:** Mean values, standard deviations (top) and correlation coefficients (bottom) of lattice points [13] for the $B\rightarrow K$ form factors $f_{0,+,T}(q^2)$

$q^2$ ($\mathrm {GeV}$ $^2$)	17	20	23						
$f_0(q^2)$	$0.62 \pm 0.03$	$0.72 \pm 0.03$	$0.87 \pm 0.04$						
$f_+(q^2)$	$1.13 \pm 0.05$	$1.63 \pm 0.07$	$2.68 \pm 0.13$						
$f_T(q^2)$	$1.02 \pm 0.06$	$1.47 \pm 0.08$	$2.42 \pm 0.18$						

$q^2$ ($\mathrm {GeV}$ $^2$)	17	20	23	17	20	23	17	20	23
$f_0(17)$	1.00	0.93	0.73	0.58	0.48	0.23	0.13	0.010	0.048
$f_0(20)$	–	1.00	0.87	0.41	0.45	0.24	0.059	0.093	0.075
$f_0(23)$	–	–	1.00	0.28	0.34	0.32	0.010	0.046	0.058
$f_+(17)$	–	–	–	1.00	0.78	0.30	0.36	0.21	0.051
$f_+(20)$	–	–	–	–	1.00	0.71	0.22	0.26	0.20
$f_+(23)$	–	–	–	–	–	1.00	$-$0.025	0.14	0.22
$f_T(17)$	–	–	–	–	–	–	1.00	0.79	0.46
$f_T(20)$	–	–	–	–	–	–	–	1.00	0.85
$f_T(23)$	–	–	–	–	–	–	–	–	1.00

We change the parametrization of $B\rightarrow K^*$ form factors w.r.t. our previous work [28] slightly and now use the simplified series expansion (SSE) [53]

B.1 $$\begin{aligned} F_i(q^2)&= \frac{1}{1 - q^2/M_{R,i}^2} \sum _k \alpha _k^i \left[ z(q^2) - z(0)\right] ^k, \end{aligned}$$

with three parameters $\alpha _k^i$ ($k=0,1,2$) per form factor $i = V,\, A_0,A_1, T_1,T_{23}$ and two ($k=1,2$) for $i= A_{12}, T_2$. The parameters $\alpha _0^i$ correspond to $F_i(q^2 = 0)$ and the kinematic constraints

B.2

are used to eliminate $\alpha _0^{A_{12}, T_2}$, where the kinematic factor ${\mathscr {F}}_\mathrm{kin} \equiv (M_B^2 - M_{K^*}^2)/(8 M_B M_{K^*})$ depends on the *B*- and $K^*$-meson masses. The pole masses $M_{R,i}$ are set to the values given in [23].

This parametrization allows us to consistently combine available results of form factors from different nonperturbative approaches, namely LCSR’s at large recoil and lattice at low recoil and to simultaneously implement the kinematic constraints. We determine the parameters $\alpha _i^k$ in a combined fit to form-factor predictions of $V, A_{0,1,2},T_{1,2,3}$ from the LCSR [23] and of $V, A_0,A_1,A_{12},T_1,T_2,T_{23}$ from the lattice [24, 25] calculations, including their correlations. We verified that the constraint

B.3 $$\begin{aligned} A_{12}(q_\mathrm{max}^2)&= {\mathscr {F}}_\mathrm{kin}\, A_1(q_\mathrm{max}^2) \end{aligned}$$

at the kinematic endpoint $q_\mathrm{max}^2 \equiv (M_B - M_{K^*})^2$ is satisfied to high accuracy by the above constraints and thus need not be imposed explicitly. Uninformative flat priors are chosen for all $\alpha _k^i$ with ranges

B.4 $$\begin{aligned} \alpha _0^i&\in [0,\, 1] ,&\alpha _{1}^i&\in [-2,\, 2],&\alpha _{2}^i&\in [-3,\, 3]. \end{aligned}$$

The results of the LCSR form-factor predictions have been provided to us directly by the authors of [23] at $q^2 = (0.1,\, 4.1,\, 8.1,\, 12.1)\, \text{ GeV }^2$ for $V, A_{0,1,2},T_{1,2,3}$ including the $28\times 28$ covariance matrix. Similar results could have been obtained by “drawing” form factors from the correlated parameters given in [23] and ancillary files just as for $B \rightarrow K$ lattice form factors.

For the $B \rightarrow K^*$ lattice form factors this approach is not good enough as we were not able to select more than two $q^2$ values without obtaining a singular covariance. In order to fully exploit the available information, we then contacted the authors of [25] and obtained the original values of the form factors (including correlation) at various values in the interval $q^2 \in [11.9,\, 17.8]$ to which Horgan et al. fit the SSE. The covariance has a block-diagonal structure with a 48$\times$48 block for $V, A_0, A_1, A_{12}$ and a 36$\times$36 block for $T_1, T_2, T_{23}$. Having the “raw” information on form-factor values is much more reliable and future proof, as there are no issues with artificial correlation and we could one day decide to use yet another form-factor parametrization and fit it easily to these data points.

We have compared the SSE fit (B.1) with two versus three parameters and found that in the former case lattice form factors influence the fit such that form factors tend to be higher than LCSR predictions at low $q^2$ leading to a poor fit. Hence we prefer the three-parameter setup as it provides the flexibility needed to accommodate LCSR and lattice results. Means and standard deviations of that three-parameter fit are given in Table 5, we omit the correlations for the sake of brevity but are happy to provide them.

## Appendix C: Monte Carlo sampling

The marginalization of the posterior is performed with the package pypmc [54], which incorporates the algorithm presented in [55, 56] and in addition an implementation of the variational Bayes algorithm. In every analysis we first run multiple adaptive Markov chains (MCMC) in parallel through pypmc. If necessary, chains are seeded at the SM point to exclude solutions in which multiple nuisance parameters – mostly for hadronic corrections – simultaneously deviate strongly from prior expectations.

In total, there are 19 parameters $\alpha ^i_j$ to describe $B\rightarrow K^*$ form factors and most of them are strongly correlated. But it is well known that strong correlation leads to poor sampling as it can cause the random-walk Markov chains to spend an excessive amount of time in regions of low probability and thus produce spurious peaks. To mitigate this issue, we perform a fit to form-factor constraints without any experimental data and use the resulting covariance matrix to transform parameters such that the new parameters are uncorrelated.

In all but three cases, the Markov chains then give reliable results. But when we analyze scenarios with $\mathscr {C}_{S,P}^{\mathrm {}} \ne 0$ and all experimental constraints, strong correlations appear again. As a final solution, we then use importance sampling with the initial proposal function determined by a fit of a Gaussian mixture to the MCMC samples within the variational Bayes approximation [57]. As the posterior is unimodal and closely resembles a Gaussian, only a few Gaussian components are needed; i.e., three components proved optimal by the variational approximation to the model evidence.

In the most challenging run with 62 parameters, we obtain a relative effective sample size of only 0.038%. We want to have enough independent samples *N* such that the 68% region is determined with a relative precision of about 1%. As a rule of thumb, we consider the “relative error of the error” given by $1/\sqrt{2 N}$ [38, ch. 37]. In the 62D case, we compute a total of $1.1\times 10^6$ importance samples, update the proposal after every $10^5$ samples, and combine all samples [57] such that $N \approx 3500$ and the estimated relative error of the error is 1.2 % and thus good enough for our purposes.

To create the smooth marginal plots in Figs. 1, 2, 3, we apply kernel density estimation for both MCMC and importance samples using the fast figtree library [58]. In the latter case, we additionally crop 500 outliers.

The prior or posterior predictive distribution of an observable *X* within a model *M* in which $X=f(\mathbf {\theta })$ is a definite function of the parameters $\mathbf {\theta }$ and given as

C.1 $$\begin{aligned} P(X | M)&= \int \text{ d } \, \mathbf {\theta } \, P(X|\mathbf {\theta },M) P(\mathbf {\theta }|M)\nonumber \\&= \int \text{ d } \, \mathbf {\theta } \, \delta (X - f(\mathbf {\theta })) P(\mathbf {\theta }|M). \end{aligned}$$

We estimate *P*(*X*|*M*) by computing $f(\mathbf {\theta }_i)$ for every sample $\mathbf {\theta }_i \sim P(\mathbf {\theta }|M)$, then smooth as above.

## Footnotes

1 Logarithmically enhanced NLO QED corrections to $B\rightarrow X_s \bar{\ell }\ell$ [14] turn out to be non-negligible for angular observables, however, analogous corrections to $B\rightarrow K \bar{\ell }\ell$ are partially included in the event simulation of the experimental analysis [1] via PHOTOS [15]. As proposed in [16], the measurement of higher moments of the decay distribution (1.1) could give an estimate of the size of higher-order QED corrections but still admixed with contributions of $\text{ dim } \ge 8$ operators.
